# Predictors of shell size in long‐lived lake gastropods

**DOI:** 10.1111/jbi.12777

**Published:** 2016-07-21

**Authors:** Thomas A. Neubauer, Elisavet Georgopoulou, Mathias Harzhauser, Oleg Mandic, Andreas Kroh

**Affiliations:** ^1^ Geological‐Palaeontological DepartmentNatural History Museum Vienna1010ViennaAustria

**Keywords:** Ancient lakes, freshwater gastropods, lake surface area, shell size, species richness, species–area relationship

## Abstract

**Aim:**

To investigate shell size variation among gastropod faunas of fossil and recent long‐lived European lakes and discuss potential underlying processes.

**Location:**

Twenty‐three long‐lived lakes of the Miocene to Recent of Europe.

**Methods:**

Based on a dataset of 1412 species of both fossil and extant lacustrine gastropods, we assessed differences in shell size in terms of characteristics of the faunas (species richness, degree of endemism, differences in family composition) and the lakes (surface area, latitude and longitude of lake centroid, distance to closest neighbouring lake) using multiple and linear regression models. Because of a strong species–area relationship, we used resampling to determine whether any observed correlation is driven by that relationship.

**Results:**

The regression models indicated size range expansion rather than unidirectional increase or decrease as the dominant pattern of size evolution. The multiple regression models for size range and maximum and minimum size were statistically significant, while the model with mean size was not. Individual contributions and linear regressions indicated species richness and lake surface area as best predictors for size changes. Resampling analysis revealed no significant effects of species richness on the observed patterns. The correlations are comparable across families of different size classes, suggesting a general pattern.

**Main conclusions:**

Among the chosen variables, species richness and lake surface area are the most robust predictors of shell size in long‐lived lake gastropods. Although the most outstanding and attractive examples for size evolution in lacustrine gastropods come from lakes with extensive durations, shell size appears to be independent of the duration of the lake as well as longevity of a species. The analogue of long‐lived lakes as ‘evolutionary islands’ does not hold for developments of shell size because different sets of parameters predict size changes.

## Introduction

Body size is an important functional and evolutionary trait, closely interrelated with individual fitness (Maurer *et al*., 1992; Hone & Benton, 2005; Clauset & Erwin, 2008) and metacommunity structure (Woodward *et al*., 2005; Loeuille & Loreau, 2006; Damuth, 2007; Jennings *et al*., 2007; De Bie *et al*., 2012). Size changes harbour advantages and disadvantages in terms of individual survival, fecundity, mating success, development time, nutrient requirement, dispersal and prey–predation interactions (Osenberg & Mittelbach, 1989; Hone & Benton, 2005; Jenkins *et al*., 2007; De Bie *et al*., 2012). Among populations or faunas, size distribution is coupled with species richness and abundance (McClain, 2004; Jennings *et al*., 2007; White *et al*., 2007). On a larger scale, size changes affect vulnerability to ecological crises and extinction (Payne, 2005; Twitchett, 2007; He *et al*., 2010; Metcalfe *et al*., 2011).

Notable tendencies of size increase have been documented for fossil freshwater and brackish‐water gastropods. Particularly among the families Lymnaeidae and Melanopsidae, several species lineages are famous for their outstandingly large shells (e.g. Moos, 1944; Geary *et al*., 2012; Neubauer *et al*., 2013). Members of the Valencienniinae, comprising large, low patelliform lymnaeids, attain maximum shell lengths of 125 mm (Moos, 1944). Comparable examples for extraordinary large shell sizes have been reported for brackish‐water bivalves as well (Harzhauser & Mandic, 2004; Geary *et al*., 2010).

Among non‐marine systems, such an extraordinary size evolution seems to be entirely restricted to long‐lived lakes. These environments are commonly referred to as ‘evolutionary islands’ for their exceptional biogeographical and evolutionary relationships (Browne, 1981; Arnott *et al*., 2006; Wesselingh, 2007). The analogy between lakes and islands seems to encompass also developments of size. Outstanding patterns of size evolution have been recorded for island faunas. As a general rule, large species typically tend towards dwarfisms, while small species become larger, a trend termed ‘island rule’ (Lomolino, 2005; Whittaker & Fernández‐Palacios, 2007; Lomolino *et al*., 2012, 2013; but see also Meiri *et al*., 2008 for an opposing view).

The present work investigates variation in shell size among gastropod faunas of fossil and recent long‐lived (‘ancient’) lakes across Europe. Based on a dataset of over 1400 species of lacustrine gastropods from 23 lakes, we assess differences in shell size in relation to species richness, endemism and family composition of the lake fauna, as well as a lake's physiographical parameters. Since lakes are often referred to as ‘evolutionary islands’, we compare our results with size evolution in island faunas. Moreover, we test whether the observed overall pattern of size change is a phenomenon established by certain families or constitutes a general rule valid across families of different size classes. This work is the first large‐scale evaluation of shell size evolution of lacustrine animals.

## Materials and methods

### Dataset

**Figure 1:**
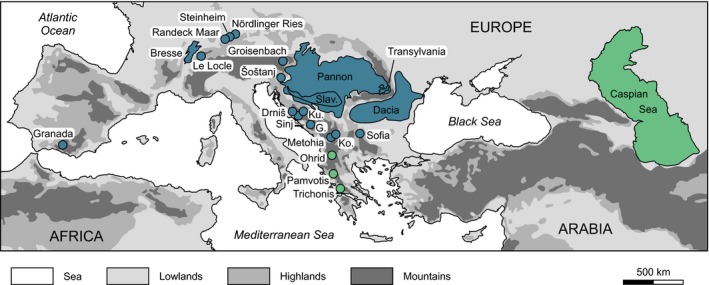
Geographical overview of the 23 Miocene to extant European lakes. The partly overlapping lake outlines result from the stratigraphic differences in the respective lakes. Palaeo‐lakes are given in blue, recent ones are marked green (for colour see online version of this article; see also Table 1 for details). G. = Gacko; Ko. = Kosovo; Ku. = Kupres; Slav. = Slavonia.

The fossil record is commonly biased towards larger species (Cooper *et al*., 2006), so we included only well‐studied faunas to ensure that small species are also covered. For the present paper, we acquired size information for faunas of 23 long‐lived European lakes (Fig. 1), comprising 1412 gastropod species deriving from 1250 localities. The dataset includes 1243 species from 19 Neogene lakes (23.03–2.588 Ma) and 185 species from four recent ones (Table 1). Only lakes with four species or more and with guaranteed continuous temporal persistence and reliable age constraints were included. For details on taxonomic and stratigraphic treatment see Neubauer *et al*. (2015a,b). Palaeo‐lake outlines for area calculation follow the reconstructions by Neubauer *et al*. (2015a) using ESRI^®^ ArcGIS^™^.

**Table 1:** Data on long‐lived lakes included in this study. Latitude and longitude refer to lake centroid. For geographical extent of the fossil and recent lakes see Figure 1. Country abbreviations follow ISO 3166‐1 Alpha‐2 code. Age ranges and durations derive from Neubauer et al. (2015a,b)

Lake	Countries covered	Max. temporal range of environment (Ma)	Latitude	Longitude	Area (km²)	Duration (Ma)	% Endemism	Distance (km)	Number of species across entire duration	Avg. beta diversity
Bresse	FR	4.5><1.5^a^	46.641	5.243	9651.04	1.80	17.19	28	64	0.540
Caspian Sea	AZ, IR, KZ, RU, TM	0.88–0	41.808	50.513	378666.31	0.88	92.39	207	92	0.528
Dacia	BG, MD, RO, UA	8.6–2.6	45.132	26.295	98730.88	6.00	56.44	30	303	0.551
Drniš	HR	15.7–15.0	43.836	16.263	24.89	0.70	16.28	12	43	0.638
Gacko	BA	15.8–15.2	43.140	18.544	39.57	0.60	0.00	60	12	0.502
Granada	ES	7.5><5.33^a^	37.110	−3.832	931.43	1.70	0.00	50	21	0.500
Groisenbach	AT	16.0><13.8^a^	47.543	15.270	22.63	0.20	91.67	40	12	0.728
Kosovo	KV	6.0–4.7	42.590	21.066	920.44	1.30	27.27	12	22	0.584
Kupres	BA	15.7–15.3	43.986	17.215	65.40	0.40	30.43	30	23	0.445
Le Locle	CH	13.5–14.0	47.074	6.779	11.38	0.50	5.56	13	18	0.413
Metohia	KV	6.04–2.588	42.501	20.544	1805.05	3.45	70.93	12	86	0.565
Nördlinger Ries	DE	15.0–13.8	48.885	10.564	438.24	1.20	16.67	5	6	0.490
Ohrid	AL, MK	1.5–0	41.037	20.716	356.37	1.50	64.71	9	68	0.409
Pamvotis	GR	0.4–0	39.663	20.884	22.70	0.40	12.00	95	25	0.481
Pannon	AT, BA, CZ, HR, HU, RO, RS, SI, SK	11.6–4.5	46.491	20.340	233485.79	7.10	74.61	55	579	0.435
Randeck Maar	DE	17.0><15.0^a^	48.576	9.526	1.08	0.30	0.00	13	4	0.607
Sinj	HR	18.0–15.0	43.695	16.682	131.80	3.00	39.66	12	58	0.556
Slavonia	BA, HR, ?HU, RS	4.5–2.0	45.404	18.765	24243.25	2.50	44.24	125	165	0.545
Sofia	BG	5.8–4.3	42.732	23.385	908.40	1.50	16.67	96	6	0.493
Šoštanj	SI	5.3><2.6^a^	46.382	15.071	13.87	0.50	66.67	67	9	0.503
Steinheim	DE	15.0–13.8	48.686	10.070	7.32	1.20	47.62	10	42	0.474
Transylvania	RO	3.8–0.8	45.840	25.751	2221.12	3.00	53.85	30	78	0.579
Trichonis	GR	2.6–0	38.559	21.552	94.83	2.60	13.04	3	23	0.549

a Precise age ranges missing (in those cases, duration is mainly estimated by available sedimentation rates).

### Size measures

Where possible, size was taken from the species’ original descriptions to avoid biases from misidentifications. These papers are the source for most of the size data because the majority of the species is endemic to single lakes and has not been described again in detail. Where the original description was unavailable to us or does not contain the required information, size was gathered from other sources that are considered taxonomically reliable. We had to exclude 22 species, for which no size information was available to us, corresponding to 1.6% of the total number of species, from the analyses.

Shell size was captured as height and width. In general, the maximum value per measure found in the literature was always recorded to exclude immature specimens. Only in rare cases is more than a single measurement available for a species, which is why we could not provide mean sizes or size ranges. In addition, the accuracy of the measurements varies considerably, usually declining with increasing size. However, no strong bias is expected from this as we used log_10_‐transformed data. To obtain a single value for the statistical analyses, we applied the estimator *S* as used by Huang *et al*. (2015) and calculated as follows:

$$S=\sqrt{h\cdot w}$$

This value reflects the length of a square that has the same area as the rectangle formed by height *h* and width *w*. Thereby, it contains the information of both height and width, facilitating the comparison between dissimilar morphologies such as planorbids (planispiral) and hydrobiids (turriform).

### Predictor variables, resampling and statistical analyses

Four size measures were used for the regression models (all log_10_‐transformed), that is, mean size (*S*_mean_), to investigate differences in the average size of the studied faunas; maximum (*S*_max_) and minimum size (*S*_min_), to assess extreme values; and size range (*S*_range_), as a measure of the covered size spectrum. We investigated potential relationships between these measures and eight lake‐ and fauna‐specific parameters, that is, latitude and longitude of lake centroid, lake surface area, closest distance to neighbouring lake, species richness of the lake's gastropod fauna, its degree of endemism (i.e. relative abundance of single‐lake endemics) and average family‐level beta diversity, using linear and multiple regressions. Individual contributions of the variables of the multiple regression models were measured with hierarchical partitioning. As we include only well‐studied and sampled lakes, the given species richness values are considered to be fairly representative of the actual diversity. Distance to the closest coevally present lake was chosen to provide a rough measure for isolation. In some cases, the distances had to be approximated because exact outlines of palaeo‐lakes are rarely available, and Europe's geodynamic development during the Neogene altered basin configuration. Differences in family composition among the lakes, which might affect the size distribution of a fauna, were evaluated using the Beta‐Jaccard (β_jac_) dissimilarity measure for pairwise comparisons (Baselga *et al*., 2013). To obtain a single value for the regression models, all pairwise values per lake were averaged, representing the mean family‐level difference. In order to provide a reliable estimate of endemism, a dataset of *c*. 200 Neogene and over 1100 recent lake faunas was evaluated (Neubauer *et al*., 2015a,b,c; Georgopoulou *et al*., 2016). We assessed normality using Shapiro–Wilk tests and Q‐Q plots. Prior to the analysis, we tested for multicollinearity among the predictor variables by calculating the variance inflation factor (VIF). The presence of multicollinearity, that is, significant correlation between two of more predictor variables, may have detrimental effects on the estimated regression parameters (for details on the method and results see Appendix S1 in Supporting Information).

**Figure 2:**
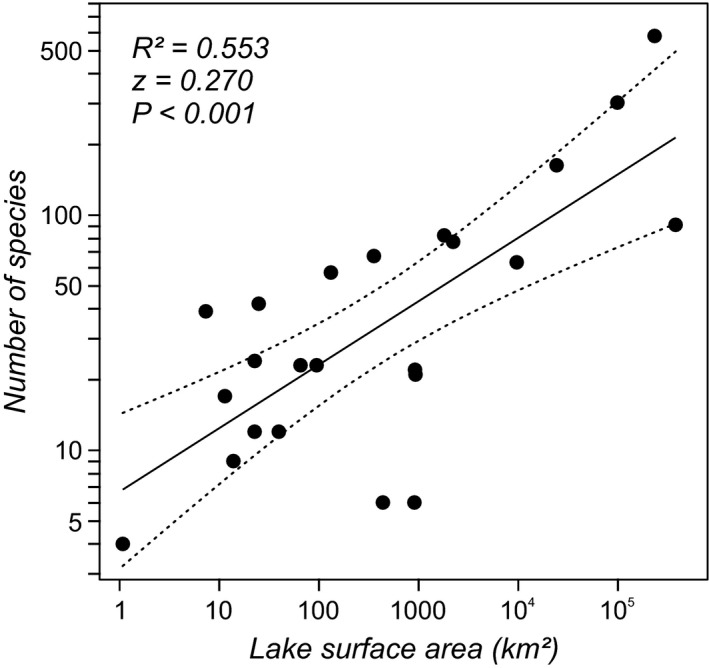
Species–area relationship for the 23 Miocene to recent European lake faunas. The correlation is based on the number of measured species, which largely coincides with the total species richness.

The investigated lakes show a strong species–area relationship, which is not unexpected but reflects their character as continental islands (Wesselingh, 2007; Neubauer *et al*., 2015a,c; Fig. 2). Similarly, the degree of endemism is correlated with species richness (*R* = 0.514, *P* = 0.012). We applied resampling to determine whether any observed correlation between size measures and area and endemism, respectively, are because of these relationships. We randomly resampled 25% of the species of each lake (minimum two species to calculate ranges), re‐calculated the respective size measures and repeated the linear regressions with area and degree of endemism. The procedure was repeated 1000 times; the *P*‐value of the test was the proportion of resampled slopes that were greater than the observed slope (Manly, 1997; Roy & Martien, 2001). The null model here is that the observed correlations are independent of species richness.

We also tested for differences among relationships of size measures with selected significant parameters among different gastropod families. Clades with only one species per lake were excluded given the lack of a size range. Only families present in more than five lakes were considered. Given the restricted datasets, we applied linear regressions for each parameter.

To assess how the factor time affects shell size evolution, we tested for linear relationships between shell size measures of a fauna at a distinct stratigraphical horizon in a lake and the age of the lake at that time. Only stratigraphically well‐resolved lakes were included (see Table S1.7 in Appendix S1). To test for the strength of the relationship of size measures with species richness at different levels, we repeated the linear regressions using the subdivided faunas where applicable. Additionally, we tested for a potential linear dependence between individual species longevity and size. For the latter analysis, the minimum and maximum stratigraphical ages of localities bearing the respective species were gathered from the literature to obtain a wider perspective (see Neubauer *et al*., 2015b). Localities with a dating uncertainty > 3 Myr were excluded from both analyses. We assessed normality of species longevity and shell size using Shapiro–Wilk tests and Q‐Q plots.

Information on the phylogenetic relationships among lineages of Valencienniinae referred to in the discussion are derived from Moos (1944). Data on the *Melanopsis*‐lineage is based on the morphometric study by Neubauer *et al*. (2013). Since real ages of clade nodes are mostly unknown, we use clade ranks instead: they reflect the number of measurable speciation events between first and last species in the lineage and thus the evolutionary position of a species within a clade (see, for example, Knouft & Page, 2003).

All statistical analyses were computed in R 3.1.3 (R Core Team, 2015) using packages ‘betapart’ 1.3 (Baselga *et al*., 2013), ‘HH’ 3.1‐14 (Heiberger, 2015), ‘hier.part’ 1.0‐4 (Walsh & Mac Nally, 2013), ‘moments’ 0.14 (Komsta & Novomestky, 2015) and ‘sampling’ 2.7 (Tillé & Matei, 2015).

## Results

**Figure 3:**
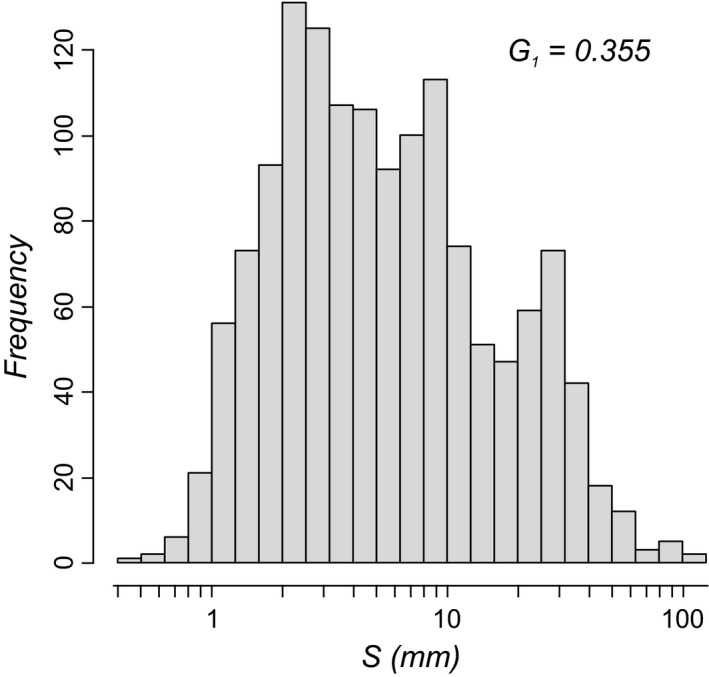
Size distribution for Miocene to recent European lacustrine gastropods in the long‐lived lakes, showing a weakly right‐skewed pattern (*G*_*1*_ = skewness).

The frequency distribution of shell sizes exhibits a non‐normal, weakly right‐skewed pattern (Shapiro–Wilk test: *W* = 0.977, *P* < 0.001; Skewness: *G*_1_ = 0.355; Fig. 3). Right‐tailed distributions are typical for many other taxa as well (Maurer *et al*., 1992; Knouft & Page, 2003; Clauset & Erwin, 2008), whereas communities on small landmasses are expected to show less positively skewed body size distributions (Maurer *et al*., 1992; Marquet & Taper, 1998).

The mean family‐level differences as evaluated with the Beta‐Jaccard (β_jac_ *)* dissimilarity analysis are summarized in Table 1 (for full matrix see Table S2.14 in Appendix S2). The Shapiro–Wilk tests indicate normal distribution for all variables used in the multiple regressions except maximum size (*P* = 0.035), size range (*P* = 0.031) and longitude (*P* = 0.010) (see Table S1.1 in Appendix S1). We still chose to include them in the regression models because the Q‐Q plots indicate that most cases match the expected distribution (see Fig. S1.1 in Appendix S1).

**Figure 4:**
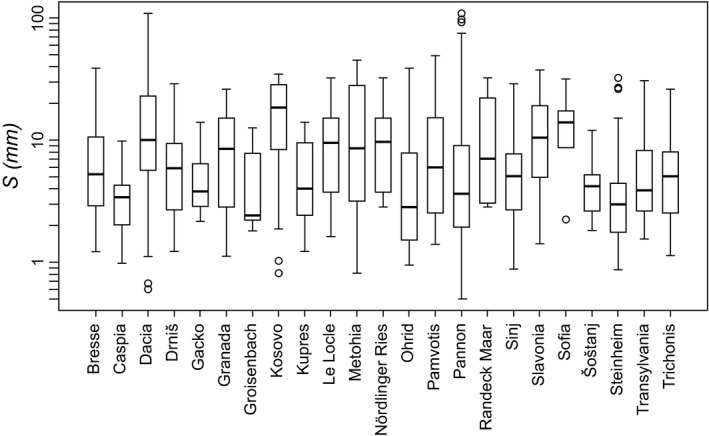
Boxplots showing size distributions within Miocene to recent long‐lived European lake faunas. Note the extraordinary wide size ranges within faunas of the lakes Pannon and Dacia, holding the largest as well as smallest species.

Size ranges and mean, maximum and minimum sizes of lacustrine faunas vary considerably across the studied lakes (Fig. 4). The broadest size ranges, as well as the largest and smallest species, occur in the two big, long‐lived lakes Pannon and Dacia. Even among faunas of similar size ranges, mean size may diverge strongly, reflecting the various faunal compositions.

Given the lack of multicollinearity (VIF values < 3), all explanatory parameters were included in the models (see Table S1.2 in Appendix S1). The models for size range, maximum size and minimum size are all significant, with the combination of variables explaining between 35.4% and 72.5% of the variation (Table 2). In all three models, species richness proves to be the most important contributor, followed by surface area. The relationships of both variables with size measures indicate that with increasing values, expanding size ranges can be expected, variably related to increasing maximum size or decreasing minimum size. The model with mean size is not significant (*P* = 0.379).

**Table 2:** Results of the multiple and linear regression models. MR = multiple regression; SE = Standard error; I = individual contribution of the predictor variables, determined by hierarchical partitioning. The two highest individual contributions for each multiple regression as well as *R²*‐values from significant linear regressions are marked in bold

Size measure	Multiple regression								Linear regression					
	*R* ^2^_adj_	*F*	*P*	Variables	Slope MR	SE	*P*	I (%)	Slope *b*_1_	SE	Intercept *b*_0_	SE	*P*	*R* ^2^
Size range	0.381	2.93	0.038	Area	0.039	0.053	0.476	**18.88**	0.068	0.038	1.271	0.113	0.083	0.137
				Beta diversity	−0.213	0.667	0.754	3.62	−0.772	0.808	1.856	0.423	0.340	0.042
				Distance	−0.146	0.114	0.220	10.55	−0.122	0.127	1.622	0.189	0.348	0.042
				Endemism	−0.004	0.002	0.134	9.56	−0.001	0.002	1.470	0.097	0.788	0.004
				Latitude	0.013	0.016	0.443	1.80	0.004	0.019	1.276	0.837	0.838	0.002
				Longitude	−0.004	0.006	0.554	5.96	−0.003	0.006	1.507	0.122	0.591	0.014
				Species richness	0.359	0.141	0.022	**49.52**	0.286	0.092	1.011	0.150	0.006	**0.280**
Maximum size	0.354	2.73	0.049	Area	0.037	0.051	0.472	**19.10**	0.064	0.035	1.311	0.106	0.088	0.133
				Beta diversity	−0.200	0.639	0.759	3.63	−0.711	0.758	1.850	0.403	0.359	0.040
				Distance	−0.129	0.109	0.254	9.88	−0.109	0.119	1.630	0.178	0.372	0.038
				Endemism	−0.003	0.002	0.141	9.74	−0.001	0.002	1.495	0.091	0.788	0.004
				Latitude	0.014	0.015	0.392	2.55	0.005	0.018	1.242	0.784	0.768	0.004
				Longitude	−0.004	0.006	0.559	6.25	−0.003	0.006	1.532	0.114	0.578	0.015
				Species richness	0.328	0.135	0.028	**48.86**	0.262	0.088	1.075	0.142	0.007	**0.299**
Minimum size	0.725	9.29	0.000	Area	0.009	0.025	0.714	**15.13**	−0.071	0.024	0.286	0.072	0.007	**0.296**
				Beta diversity	0.172	0.314	0.593	1.70	0.405	0.577	−0.113	0.307	0.490	0.023
				Distance	0.091	0.054	0.110	4.63	0.017	0.092	0.076	0.136	0.852	0.002
				Endemism	−0.002	0.001	0.157	11.70	−0.003	0.001	0.212	0.062	0.033	**0.199**
				Latitude	0.018	0.008	0.028	8.54	0.016	0.013	−0.626	0.570	0.216	0.072
				Longitude	0.002	0.003	0.486	2.63	−0.005	0.004	0.189	0.084	0.237	0.066
				Species richness	−0.291	0.066	0.001	**55.66**	−0.300	0.044	0.560	0.071	0.000	**0.692**
Mean size	0.049	1.162	0.379	Area	0.076	0.046	0.123	**25.38**	0.017	0.029	0.880	0.085	0.552	0.017
				Beta diversity	0.370	0.587	0.538	2.40	0.029	0.586	0.910	0.312	0.962	0.000
				Distance	−0.069	0.100	0.505	6.05	−0.064	0.091	1.016	0.136	0.489	0.023
				Endemism	−0.004	0.002	0.082	**48.89**	−0.003	0.001	1.024	0.064	0.062	0.156
				Latitude	0.008	0.014	0.582	2.12	0.000	0.013	0.906	0.595	0.974	0.000
				Longitude	−0.002	0.006	0.707	10.48	−0.004	0.004	1.005	0.085	0.295	0.052
				Species richness	−0.020	0.124	0.877	4.67	−0.005	0.079	0.933	0.128	0.948	0.000

**Figure 5:**
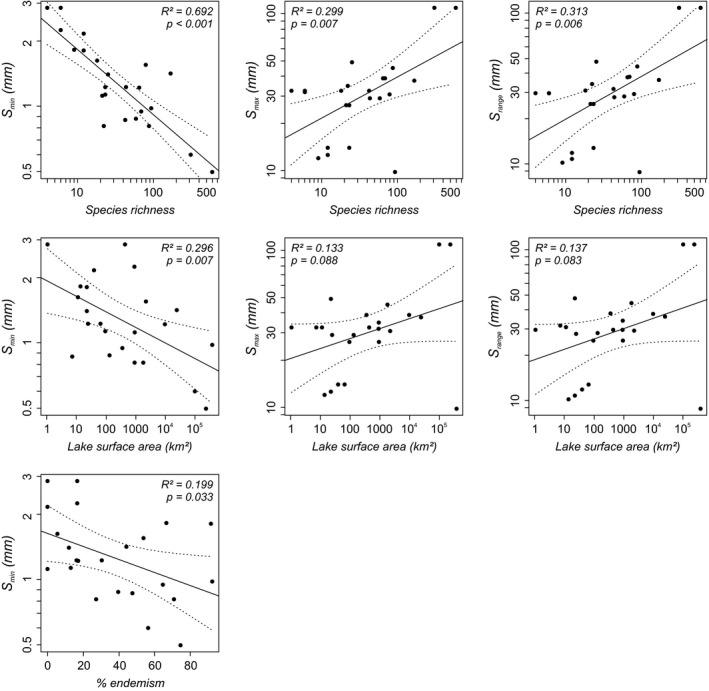
Plots of all significant linear regression models between size measures and predictor variables (all log_10_‐transformed) across all Miocene to recent European lacustrine gastropod taxa. *S*_min_ = minimum shell size per fauna; *S*_max_ = maximum shell size per fauna; *S*_range_ = shell size range per fauna; *S*_mean_ = mean shell size per fauna. Dashed lines indicate 95% confidence intervals.

The linear regressions between all variables and size measures yield similar results as the multiple regressions (Fig. 5, Table 2), confirming species richness and area as the most important predictors for size range, maximum and minimum size. However, the regressions between lake surface area and maximum size and size range, respectively, are hardly significant (*P* = 0.088 and *P* = 0.083), which reflects the exceptionally small‐sized fauna of the Caspian Sea (Fig. 5). Excluding the Caspian fauna, both regressions are highly significant (maximum size: *R²* = 0.426, *P* < 0.001; size range: *R²* = 0.424, *P* = 0.001). The strong correlation between minimum and maximum size and size range with species richness also persists when using the temporally subdivided faunas (as used for the regression with lake age; see Tables S1.10 and S1.12 in Appendix S1). Additionally, the degree of endemism yields a low relationship with minimum size. Resampling analyses reveal no significant effects of species richness on the observed correlations with area and endemism (all *P* > 0.05; see Table S1.13 in Appendix S1).

The linear regressions between size measures and species richness on the family level reveal similar results across different groups (see Table S1.3 and Fig. S1.2 in Appendix S1) irrespective of the overall size of the clade. Although *R*² values vary across different families and size measures, comparable results are obtained for clades of generally small size (Bithyniidae, Hydrobiidae, Neritidae and Valvatidae) and those comprising large species and wide size ranges (Lymnaeidae and Melanopsidae). The Planorbidae, comprising equally small and large species, show rather low correlations between size measures and richness. Similar as for species richness, the regressions between size measures and lake surface area exhibit moderate to high values across the Hydrobiidae, Neritidae, Valvatidae, Lymnaeidae and Bithyniidae (see Table S1.4 and Fig. S1.3 in Appendix S1).

The variables for the regression of size onto species longevity are not normally distributed, but the Q‐Q plots indicate that most of the cases fit the expected distribution (see Table S1.5 and Fig. S1.4 in Appendix S1). The linear regression yields a significant but minor association (*R*²_adj_ = 0.033, *P* < 0.001; see Table S1.6 and Fig. S1.5 in Appendix S1). None of the regressions between size measures and lake age is significant (see Tables S1.8 and S1.9 in Appendix S1).

**Figure 6:**
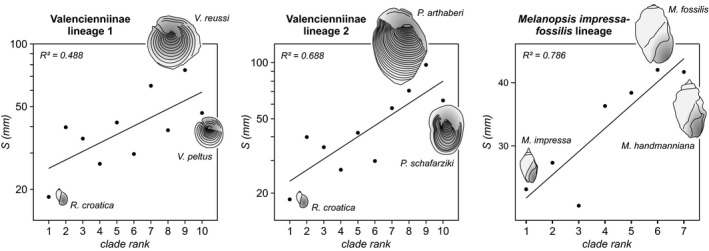
Shell size evolution in three species lineages endemic to Lake Pannon. Phylogenetic relationships for Valencienniinae lineages follow Moos (1944); size data for the melanopsid lineage derive from Neubauer *et al*. (2013). Note that in all lineages the final stage involves a size decrease. Schematic drawings of phylogenetic starting and end point as well as the largest representative are provided for each lineage (specimens to scale within each plot). *M*. = *Melanopsis*;* P*. = *Provalenciennesia*;* R*. = *Radix*;* V*. = *Valenciennius*.

The phylogenetic lineages of Valencienniinae and Melanopsidae all exhibit significant size increases over evolutionary time, while showing considerable variation on smaller scales (Fig. 6).

## Discussion

Lakes are commonly considered as islands in terms of faunal evolution and biogeographical relationships (Browne, 1981; Arnott *et al*., 2006; Wesselingh, 2007). This comparison is particularly often applied to long‐lived lakes, which accommodate highly diverse faunas (e.g. Martens, 1997; Schön & Martens, 2004; Wesselingh, 2007; Harzhauser & Mandic, 2008; Hauffe *et al*., 2011; Wagner *et al*., 2012; Neubauer *et al*., 2013). Both in recent and fossil representatives, intralacustrine radiations produced diversities far above the common average and high levels of endemism (e.g. Schön & Martens, 2004; Wesselingh, 2007; Harzhauser & Mandic, 2008; Neubauer *et al*., 2015a,c). Islands are well known for their unique endemic developments related to body size. As a general rule, small species tend to increase in size (island gigantism) and larger species become smaller (island dwarfism); but see also Meiri *et al*. (2008). Both are frequent phenomena, demonstrated for numerous animal groups (Lomolino, 2005; Whittaker & Fernández‐Palacios, 2007; Lomolino *et al*., 2013 and references therein).

Size evolution in island species is considered to converge on a size optimal for ecological strategies in dependence on characteristics of the island such as time in isolation, latitude, island area, and species interactions, that is, competition and predation (Lomolino, 2005; Lomolino *et al*., 2012, 2013). Regarding lakes as islands, we assessed the developments of shell size in lacustrine gastropods in a comparable manner. Our results indicate that a different combination of parameters is related to shell size trends. Species richness and lake area are significant predictors for gastropod body size in long‐lived lakes, while geographical isolation, latitude and temporal existence of a lake apparently play minor roles. With rising diversity and lake surface area, maximum size increases and minimum size decreases (Fig. 5, Table 2). The Caspian Sea seems to be an exception to the rule as it is characterized by an exceptionally small‐sized fauna in relation to its large surface area. Possibly, large gastropods were out‐competed there by the highly diverse bivalve fauna comprising numerous large Dreissenidae and Lymnocardiidae (Logvinenko & Starobogatov, 1968). The individual linear model of minimum size with degree of endemism is marginally significant and indicates a weak relationship.

### What drives shell size evolution?

The selective forces underlying the trend towards expanding size range are difficult to infer from the mostly fossil data. The theoretical optimal size depends highly on characteristics of the environment and ecological interactions, such as competition and predation (Lomolino *et al*., 2012). Size changes variably impact individual survival, fecundity, mating success, development time, requirement of nutrients and prey–predation interactions (e.g. Osenberg & Mittelbach, 1989; Kingsolver & Pfennig, 2004; Hone & Benton, 2005; Metcalfe *et al*., 2011). On the larger scale, Heim *et al*. (2015) suggest basic body plan and ecological life mode as driving factors favouring overall increasing size in marine animals rather than competitive advantages. Size decrease in the fossil record is, in turn, commonly related to lowered vulnerability to ecological crises and extinction (Payne, 2005; Twitchett, 2007; He *et al*., 2010; Metcalfe *et al*., 2011). The only detailed study on size variation in a fossil brackish‐water gastropod lineage is provided by Geary *et al*. (2012). They demonstrated successive size increase in a melanopsid species lineage from Lake Pannon, interpreted as greater individual longevity. Escape from predators, avoidance of resource competition, and especially increased fecundity are discussed as potential selective agents. Similarly, Lomolino *et al*. (2012, 2013) indicated for island populations that body size variation in both large and small species is influenced by ecological interactions, whereas pure size decrease is mainly a response to climatic, geographical and physiographical variables.

The positive relationship between shell size and lake surface area compares with developments on islands. Studies on insular species have yielded diverse results with respect to island area, variably demonstrating decreasing body size range (Marquet & Taper, 1998) and increasing maximum body size (Maurer *et al*., 1992) for islands of decreasing area. All studies, however, indicate that mean size tends to converge on a hypothetical optimum (Lomolino, 2005; Lomolino *et al*., 2012, 2013). Area is positively coupled with resource availability and diversity of habitats, predators and competitors (Lomolino, 2005; Lomolino *et al*., 2012). That relationship is strongly linked to the scaling of individual space requirements and its impact on population survival in environments of different area (Marquet & Taper, 1998). With increasing lake size, resource availability and habitat diversity is expected to rise as well, facilitating the coexistence of more species with different resource requirements (Marquet & Taper, 1998 and references therein). Possibly, the expansion of size ranges towards particularly small or large morphologies is a way to avoid competition for nutrients or for ecological niches in general. In several deposit‐feeding marine snails, resource‐partitioning for different particle sizes has been reported to be correlated with animal size (Fenchel *et al*., 1975; Whitlatch & Obrebski, 1980).

The strong relationship between shell size and species richness must be interpreted cautiously. In an environment of about constant size and habitat diversity, an increasing number of species would probably result in greater interspecific competition and might account for evolutionary adaptations. Diversity in our case, however, is strongly positively correlated with lake area, which is why this explanation is hardly supported. Increasing size range and maximum size and decreasing minimum size, that is, an expansion of the size distribution of a fauna in both directions, is more probable the more species a fauna holds. Hence, the evolution of particularly large and small species might merely be a matter of probability, depending on overall species richness. Nevertheless, we show that the relationships between size and other predictor variables are robust when resampling methods are applied to alleviate the effect of species richness.

Size variation as a response to predation is weakly supported by previous studies (Geary *et al*., 2012). Predation pressure has been considered a main trigger for the morphological evolution in a hydrobiid clade in extant Lake Ohrid (Schreiber *et al*., 2012), whereas its effect on shell size evolution has not been explored so far. Similarly, predation pressure by crabs apparently promotes morphological evolution in a *Theodoxus* species lineage in the latest Miocene of the Thessaloniki area, while shell size remained unaffected (Rust, 1997).

The response to selective agents certainly varies among different faunas and lakes, depending on ecological setting and community structure (Lomolino *et al*., 2012, 2013). Unfortunately, these considerations cannot be tested individually since most of the studied environments no longer exist.

### Other factors related with size

Because of varied evolutionary processes, one might also expect differing developments of shell size between species that evolved within a lake by intralacustrine speciation and those transported into the lake. While the degree of endemism does not seem crucial for the evolution of particularly large sizes, it does affect the minimum size of a fauna (Fig. 5). The negative relationship between minimum size and degree of endemism indicates that increasing endemism yields an averagely smaller fauna. This correlation is strongly influenced by the Caspian Sea and Lake Groisenbach, which have highly endemic faunas (92.3% and 91.7% respectively) that comprise mostly small species. On the one hand, this result may imply that those species evolved through intralacustrine speciation are tendentially smaller. Another, more likely option is that a higher number of small species evolved within the lakes than large ones, shifting minimum size towards lower values. Despite this correlation for the overall gastropod fauna, no significant relation between endemism and minimum shell size on the family level was detected (see Fig. 3.6 in Appendix S3).

The absence of a relationship between size measures and latitude is not surprising because only a narrow latitudinal range is covered by the studied lakes. Across a variety of organisms, size has been shown to increase with higher latitudes, reflecting better heat retention because of a lowered surface‐volume ratio, a pattern generally referred to as Bergmann's Rule (Meiri, 2011; Berke *et al*., 2013). Inclusion of more data to test for a possible dependence is unfortunately hampered by the limited availability of outcrops. Because of erosion by advancing and retreating ice shields during the Ice Ages, no surface outcrop with Neogene sediments is preserved north of *c*. 52° N. Likewise, information on southernmost European gastropod faunas is limited (Neubauer *et al*., 2015a,b). In addition, a clear latitudinal pattern cannot be expected because the lakes come from different time slices with different climatic regimes.

The absence of an association of shell size with isolation, being a measure of immigration selection on islands (Whittaker *et al*., 2001, 2014; Lomolino, 2005), may root in the limited dispersal possibilities for many groups of continental aquatic gastropods. While many freshwater caenogastropods and viviparids require good hydrological connectivity of surface waters for their dispersal (see Van Bocxlaer *et al*., 2011 and references therein), truncatelloid and pulmonate snails largely rely on passive dispersal, commonly via waterfowl or post‐larval drift (Kappes & Haase, 2012; van Leeuwen *et al*., 2012, 2013). Inferences about dispersal mechanisms for fossil freshwater gastropods are rare, but indicate the presence of similar modes at that time already (Harzhauser *et al*., 2016). The successful introduction or active immigration into other continental aquatic systems depends on abiotic and biotic preferences and limitations, so the effective faunal exchange is probably limited and not necessarily dependent on geographical distance. Moreover, there is a crucial difference between lakes and islands in terms of isolation. Patterns of body size evolution as well as diversification events in island species are commonly discussed with reference to their mainland relatives (Lomolino, 2005; Whittaker & Fernández‐Palacios, 2007; Stuart *et al*., 2012). Lake faunas, in contrast, represent mixed entities that cannot be assigned to a common origin, but rather comprise an accumulation of species variably deriving from intralacustrine speciation and introduction from different origins.

### Generality of the pattern at different levels

The relationship between size and species richness and area, respectively, varies across different families, but seems independent of size class. It applies equally to the small‐sized Hydrobiidae (mean *S* = 2.9 mm) and the moderate‐ to large‐sized Lymnaeidae (mean *S* = 26.9 mm). This fact also explains why differences in the lakes’ family compositions scarcely affect changes of shell size. No significant association was detected for the family Viviparidae, which nonetheless constitutes important parts of the studied faunas. Viviparidae seem relatively size‐stable through time. Conspicuous morphological radiations have been reported for viviparids across several long‐lived Neogene lakes, all of which involve changes of shape and sculpture rather than size (e.g. Willmann, 1981; Lubenescu & Zazuleac, 1985; Mandic *et al*., 2015).

### Within‐lake patterns

Size evolution through time is commonly discussed with reference to Cope's Rule, that is, the tendency for organisms in evolving lineages to increase in size over time (Hone & Benton, 2005). While numerous theoretical and empirical studies support this hypothesis (Alroy, 1998; Kingsolver & Pfennig, 2004; Hone *et al*., 2005; Novack‐Gottshall & Lanier, 2008; Heim *et al*., 2015), a considerable amount of works argues against it (e.g. Jablonski, 1997; Knouft & Page, 2003; Harries & Knorr, 2009). Gastropod lineages in long‐lived lakes differ considerably from marine and terrestrial groups because of their relatively limited temporal existence. With the disappearance of the environment, the often highly specialized species and thus the lineages become extinct too (see, for example, Harzhauser & Mandic, 2004). Only few cases of successful immigration to other lakes are known (Wesselingh, 2007).

Unfortunately, little is known about the phylogenetic relationships among the studied gastropods, which is why an inference about the validity of Cope's Rule in lacustrine snails cannot be made from our data. Figure 6 shows size changes in the few species lineages derived from the literature where the phylogenetic relationships are known. While the trend towards an overall increasing size is significant for all three lineages (corresponding to Cope's Rule), each of them involves notable size variation, particularly in the case of the Valencienniinae. Similarly, the faunas of the lakes Pannon and Metohia reach their maximum and minimum size as well as their widest size ranges in their early phases (see Table S1.7 in Appendix S1). Hence, shell size evolution involves a considerable degree of variation over time, explaining the absence of a relationship of size measures with lake age.

## Conclusion

Species richness and lake surface area are the best predictors of shell size of gastropods from long‐lived lakes. With both variables increasing, shell size range expands, maximum size increases and minimum size decreases. Moreover, minimum shell size is weakly related to the degree of endemism of a lake's entire gastropod fauna, suggesting that a higher number of small species originate from intralacustrine speciation. Size is independent of the longevity of a species or lake age. This is an important conclusion as the most outstanding and attractive examples for size evolution in lacustrine gastropods (the Valencienniinae) derive from lakes Pannon and Dacia, and have therefore often been related to the extensive durations of these environments. We provide selected examples that indicate that size evolution is not necessarily linear but involves variation on smaller temporal scales, explaining the lacking correlation with time. Resampling analyses substantiated that the detected correlations are not driven by differences in diversity among the lakes. Moreover, the strong relationships between shell size and species richness and area, respectively, are consistent across gastropod families. This suggests a general rule unaffected by differences in ontogenetic development.

Given the unavailability of ecological data, we could only hypothesize about the underlying processes driving the patterns observed. Long‐lived lake environments are famous for their unique internal evolutionary processes and ecological interactions, which differ conspicuously from marine and terrestrial settings and thus may have a distinctive impact on size evolution. Broader future investigations should aim at inferring causal processes and should reveal whether the pattern is restricted to lacustrine gastropods or a phenomenon typical for inhabitants of long‐lived lakes.

## Data accessibility

All data used for this study are available from the Pangaea database: http://doi.pangaea.de/10.1594/PANGAEA.858575.

## Biosketch

**Thomas A. Neubauer** is a palaeobiologist and PostDoc at the Natural History Museum Vienna. He and the whole FreshGEN team are interested in reconstructing large‐scale patterns of biodiversity and biogeography of Miocene to Recent European freshwater gastropod faunas.

Author contributions: M.H. and O.M. developed the initial design for the study; A.K. and T.A.N. developed the database; T.A.N., E.G., M.H. and O.M. compiled the data; T.A.N., E.G. and A.K. analysed the data; all authors contributed to the interpretations; T.A.N. wrote the manuscript.

## Supporting information


**Appendix S1** Basic statistics, linear regressions and resampling.

**Appendix S2** Beta‐Jaccard dissimilarity measure for pairwise comparisons.

**Appendix S3** Minimum size versus endemism for gastropod families.
