# Supplementary material for: Predictors of shell size in long‐lived lake gastropods
Source: J Biogeogr. 2016 Jul 21;43(10):2062–74. doi: 10.1111/jbi.12777 (PMC5042061; doi:10.1111/jbi.12777)
Supplement: Supplementary file 1 — Appendix S1 Basic statistics, linear regressions and resampling. [file JBI-43-2062-s001.docx]

*Journal of Biogeography*

**SUPPORTING INFORMATION**

**Predictors of shell size in long-lived lake gastropods**

Thomas A. Neubauer*, Elisavet Georgopoulou, Mathias Harzhauser, Oleg Mandic, Andreas Kroh

**Appendix S1**

Additional results and plots for basic statistics, linear regressions and resampling.

***1. Multiple regression***

**Table S1.1** Shapiro-Wilk normality test

| **Parameter/size measure** | ***W*** | ***P*** |
| --- | --- | --- |
| Maximum size | 0.9070 | 0.03539 |
| Mean size | 0.9628 | 0.5231 |
| Minimum size | 0.9811 | 0.9247 |
| Size range | 0.9044 | 0.03118 |
| % Endemism | 0.9242 | 0.082 |
| Average beta diversity | 0.9599 | 0.4619 |
| Latitude | 0.9625 | 0.5153 |
| Longitude | 0.8796 | 0.009838 |
| log(Distance) | 0.9698 | 0.6832 |
| log(Species richness) | 0.9764 | 0.8367 |
| log(Surface area) | 0.9542 | 0.357 |

**Fig. S1.1** QQ-plots for non-normal parameters


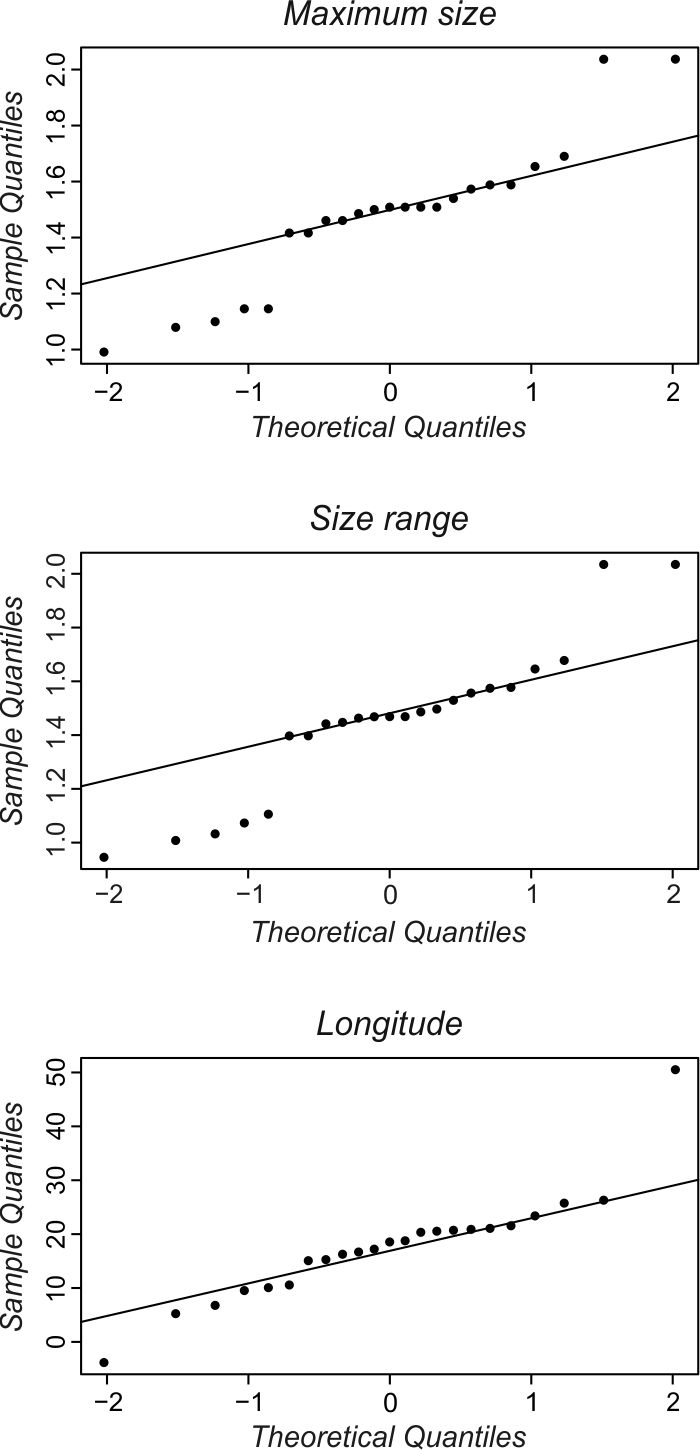


**Test for multicollinearity**

Biological data usually involve a certain degree of correlation among predictor variables, which can severely bias the estimation of model parameters (Quinn & Keough, 2002; Legendre & Legendre, 2012). Looking at correlations only among pairs of predictors, however, is limiting. Therefore, we tested for multicollinearity among our eight predictor variables by calculating the variance inflation factor (VIF). As a rule of thumb, VIF values greater than ten indicate the presence of multicollinearity (Quinn & Keough, 2002); some authors even consider VIF values above five evidence of collinearity (Heiberger & Holland, 2004).

**Table S1.2** Variance inflation factor (VIF) for the predictor variables used in the regression analyses

| **parameter** | **VIF** |
| --- | --- |
| % Endemism | 2.127094 |
| Average beta diversity | 1.105907 |
| Latitude | 1.229627 |
| Longitude | 1.877293 |
| log(Distance) | 1.305364 |
| log(Species richness) | 2.690524 |
| log(Surface area) | 2.868213 |

Given the lack of multicollinearity among the predictor variables (VIF < 5), we included all of them in the regression models.

**REFERENCES**

Legendre, P. & Legendre, L. (2012) *Numerical Ecology*. Elsevier, Amsterdam.

Heiberger, R.M. & Holland, B. (2004) *Statistical Analysis and Data Display: An Intermediate Course with Examples in S-Plus, R, and SAS*. Springer, Heidelberg.

Quinn, G.P. & Keough, M.J. (2002) *Experimental design and data analysis for biologists*. Cambridge University Press, Cambridge.

***2. Linear regressions for nine gastropod families with species richness and area***

**Table S1.3** Results of the linear regressions with species richness

| **Size measure** | **Family** | **Slope b_1_** | **SE** | **Intercept b_0_** | **SE** | **P** | **R^2^** |
| --- | --- | --- | --- | --- | --- | --- | --- |
| Size range | Bithyniidae | 0.389 | 0.238 | -0.123 | 0.453 | 0.145 | 0.277 |
|  | Emmericiidae | 0.142 | 0.287 | 0.257 | 0.525 | 0.637 | 0.040 |
|  | Hydrobiidae | 0.661 | 0.143 | -0.576 | 0.248 | 0.000 | 0.558 |
|  | Lymnaeidae | 0.443 | 0.259 | 0.516 | 0.480 | 0.113 | 0.196 |
|  | Melanopsidae | 0.446 | 0.241 | 0.210 | 0.474 | 0.101 | 0.300 |
|  | Neritidae | 0.861 | 0.294 | -1.314 | 0.601 | 0.022 | 0.550 |
|  | Planorbidae | 0.230 | 0.219 | 0.536 | 0.356 | 0.307 | 0.050 |
|  | Valvatidae | 0.533 | 0.244 | -0.414 | 0.471 | 0.060 | 0.375 |
|  | Viviparidae | 0.067 | 0.195 | 1.173 | 0.395 | 0.744 | 0.019 |
| Maximum size | Bithyniidae | 0.189 | 0.077 | 0.647 | 0.146 | 0.044 | 0.463 |
|  | Emmericiidae | 0.065 | 0.116 | 0.815 | 0.212 | 0.597 | 0.049 |
|  | Hydrobiidae | 0.293 | 0.069 | 0.272 | 0.119 | 0.001 | 0.518 |
|  | Lymnaeidae | 0.327 | 0.173 | 0.906 | 0.322 | 0.084 | 0.229 |
|  | Melanopsidae | 0.273 | 0.110 | 0.797 | 0.216 | 0.038 | 0.436 |
|  | Neritidae | 0.082 | 0.068 | 0.836 | 0.139 | 0.268 | 0.171 |
|  | Planorbidae | 0.135 | 0.156 | 0.818 | 0.254 | 0.398 | 0.034 |
|  | Valvatidae | 0.037 | 0.056 | 0.787 | 0.109 | 0.534 | 0.050 |
|  | Viviparidae | 0.001 | 0.077 | 1.575 | 0.156 | 0.988 | 0.000 |
| Minimum size | Bithyniidae | -0.068 | 0.128 | 0.780 | 0.244 | 0.611 | 0.039 |
|  | Emmericiidae | -0.007 | 0.114 | 0.694 | 0.208 | 0.950 | 0.001 |
|  | Hydrobiidae | -0.315 | 0.060 | 0.698 | 0.104 | 0.000 | 0.618 |
|  | Lymnaeidae | -0.288 | 0.105 | 1.367 | 0.194 | 0.018 | 0.387 |
|  | Melanopsidae | -0.457 | 0.180 | 1.654 | 0.355 | 0.035 | 0.446 |
|  | Neritidae | -0.486 | 0.095 | 1.701 | 0.194 | 0.001 | 0.788 |
|  | Planorbidae | -0.208 | 0.066 | 0.509 | 0.107 | 0.005 | 0.323 |
|  | Valvatidae | -0.345 | 0.099 | 0.957 | 0.191 | 0.008 | 0.603 |
|  | Viviparidae | -0.107 | 0.095 | 1.394 | 0.194 | 0.304 | 0.174 |
| Mean size | Bithyniidae | 0.136 | 0.081 | 0.627 | 0.153 | 0.136 | 0.289 |
|  | Emmericiidae | -0.005 | 0.098 | 0.819 | 0.179 | 0.958 | 0.000 |
|  | Hydrobiidae | -0.005 | 0.046 | 0.484 | 0.079 | 0.919 | 0.001 |
|  | Lymnaeidae | 0.046 | 0.122 | 1.157 | 0.227 | 0.713 | 0.012 |
|  | Melanopsidae | -0.022 | 0.052 | 1.125 | 0.103 | 0.687 | 0.021 |
|  | Neritidae | -0.131 | 0.066 | 1.154 | 0.134 | 0.086 | 0.363 |
|  | Planorbidae | -0.100 | 0.105 | 0.792 | 0.170 | 0.351 | 0.042 |
|  | Valvatidae | -0.087 | 0.053 | 0.818 | 0.103 | 0.139 | 0.252 |
|  | Viviparidae | -0.040 | 0.043 | 1.509 | 0.087 | 0.386 | 0.127 |

**Table S1.4** Results of the linear regressions with lake surface area

| **Size measure** | **Family** | **Slope b_1_** | **SE** | **Intercept b_0_** | **SE** | **P** | **R^2^** |
| --- | --- | --- | --- | --- | --- | --- | --- |
| Size range | Bithyniidae | 0.167 | 0.076 | 0.043 | 0.273 | 0.064 | 0.409 |
|  | Emmericiidae | -0.012 | 0.099 | 0.549 | 0.330 | 0.905 | 0.003 |
|  | Hydrobiidae | 0.157 | 0.056 | 0.107 | 0.171 | 0.012 | 0.319 |
|  | Lymnaeidae | 0.100 | 0.082 | 1.032 | 0.259 | 0.246 | 0.110 |
|  | Melanopsidae | 0.097 | 0.093 | 0.739 | 0.334 | 0.328 | 0.119 |
|  | Neritidae | 0.254 | 0.088 | -0.535 | 0.347 | 0.023 | 0.546 |
|  | Planorbidae | 0.016 | 0.082 | 0.845 | 0.244 | 0.842 | 0.002 |
|  | Valvatidae | 0.169 | 0.096 | 0.033 | 0.338 | 0.116 | 0.279 |
|  | Viviparidae | -0.009 | 0.076 | 1.337 | 0.296 | 0.913 | 0.002 |
| Maximum size | Bithyniidae | 0.076 | 0.024 | 0.745 | 0.085 | 0.015 | 0.595 |
|  | Emmericiidae | 0.028 | 0.039 | 0.844 | 0.129 | 0.501 | 0.079 |
|  | Hydrobiidae | 0.076 | 0.025 | 0.557 | 0.077 | 0.007 | 0.355 |
|  | Lymnaeidae | 0.070 | 0.056 | 1.298 | 0.177 | 0.236 | 0.115 |
|  | Melanopsidae | 0.068 | 0.044 | 1.092 | 0.159 | 0.162 | 0.229 |
|  | Neritidae | -0.007 | 0.022 | 1.024 | 0.087 | 0.776 | 0.012 |
|  | Planorbidae | 0.006 | 0.058 | 1.009 | 0.173 | 0.916 | 0.001 |
|  | Valvatidae | 0.011 | 0.021 | 0.818 | 0.073 | 0.601 | 0.036 |
|  | Viviparidae | -0.018 | 0.029 | 1.644 | 0.112 | 0.552 | 0.062 |
| Minimum size | Bithyniidae | -0.026 | 0.045 | 0.738 | 0.163 | 0.591 | 0.043 |
|  | Emmericiidae | 0.038 | 0.036 | 0.564 | 0.118 | 0.329 | 0.158 |
|  | Hydrobiidae | -0.080 | 0.023 | 0.387 | 0.072 | 0.003 | 0.406 |
|  | Lymnaeidae | -0.094 | 0.030 | 1.115 | 0.094 | 0.008 | 0.459 |
|  | Melanopsidae | -0.107 | 0.074 | 1.138 | 0.267 | 0.187 | 0.207 |
|  | Neritidae | -0.142 | 0.030 | 1.257 | 0.117 | 0.002 | 0.768 |
|  | Planorbidae | -0.046 | 0.027 | 0.309 | 0.081 | 0.108 | 0.119 |
|  | Valvatidae | -0.116 | 0.040 | 0.689 | 0.143 | 0.021 | 0.505 |
|  | Viviparidae | -0.039 | 0.037 | 1.328 | 0.145 | 0.332 | 0.156 |
| Mean size | Bithyniidae | 0.053 | 0.027 | 0.702 | 0.098 | 0.092 | 0.352 |
|  | Emmericiidae | 0.016 | 0.033 | 0.760 | 0.109 | 0.639 | 0.039 |
|  | Hydrobiidae | -0.002 | 0.014 | 0.480 | 0.044 | 0.917 | 0.001 |
|  | Lymnaeidae | -0.002 | 0.037 | 1.246 | 0.117 | 0.959 | 0.000 |
|  | Melanopsidae | 0.004 | 0.018 | 1.068 | 0.065 | 0.814 | 0.007 |
|  | Neritidae | -0.057 | 0.012 | 1.101 | 0.046 | 0.002 | 0.768 |
|  | Planorbidae | -0.021 | 0.039 | 0.693 | 0.115 | 0.594 | 0.014 |
|  | Valvatidae | -0.030 | 0.020 | 0.753 | 0.070 | 0.169 | 0.222 |
|  | Viviparidae | -0.029 | 0.013 | 1.537 | 0.052 | 0.072 | 0.442 |

**Fig. S1.2** Plots of all significant linear regression models between size measures (all log_10_-transformed) and species richness across different families. *S_min_* = minimum shell size per fauna; *S_max_* = maximum shell size per fauna; *S_range_* = shell size range per fauna; *S_mean_* = mean shell size per fauna. Dashed lines indicate 95% confidence intervals.

**
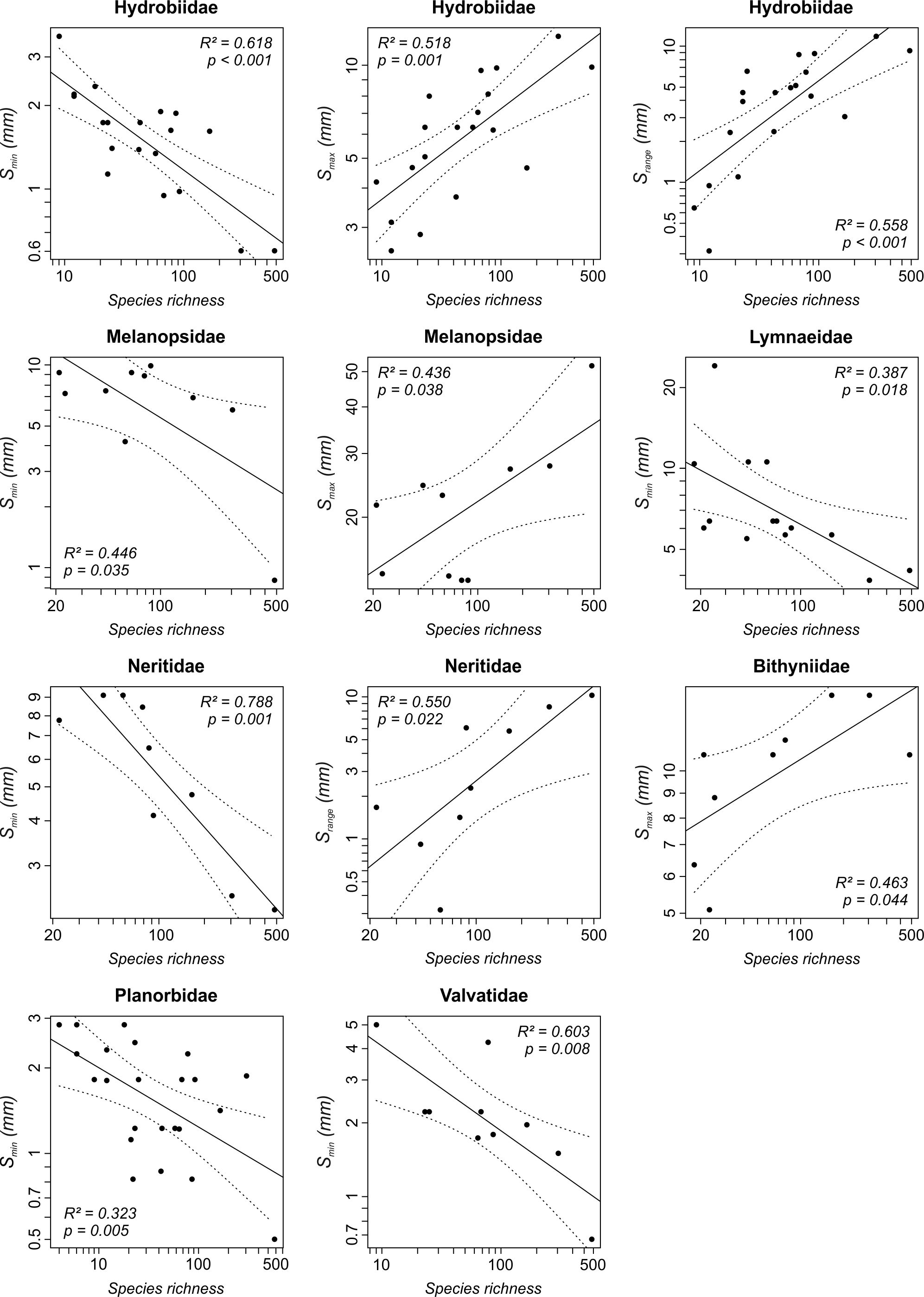
**

**Fig. S1.3** Plots of all significant linear regression models between size measures (all log_10_-transformed) and lake surface area across different families. *S_min_* = minimum shell size per fauna; *S_max_* = maximum shell size per fauna; *S_range_* = shell size range per fauna; *S_mean_* = mean shell size per fauna. Dashed lines indicate 95% confidence intervals.


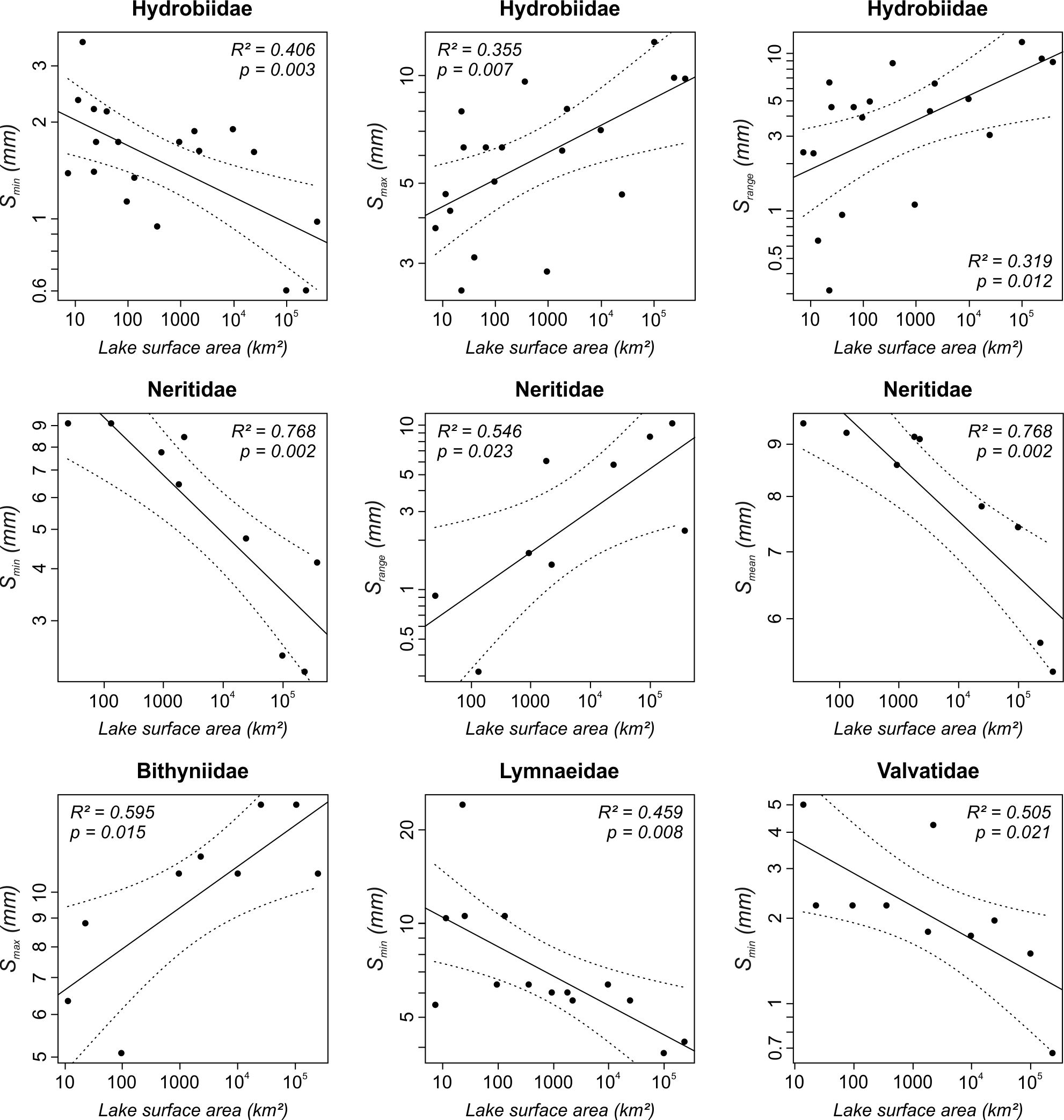


***3. Linear regressions of size with individual species longevity***

**Table S1.5** Shapiro-Wilk normality test

| **Parameter/size measure** | ***W*** | ***P*** |
| --- | --- | --- |
| log(S) | 0.977 | 6.64E-14 |
| log(Species longevity) | 0.964 | 2.20E-16 |

**Fig. S1.4** QQ-plots for non-normal parameters


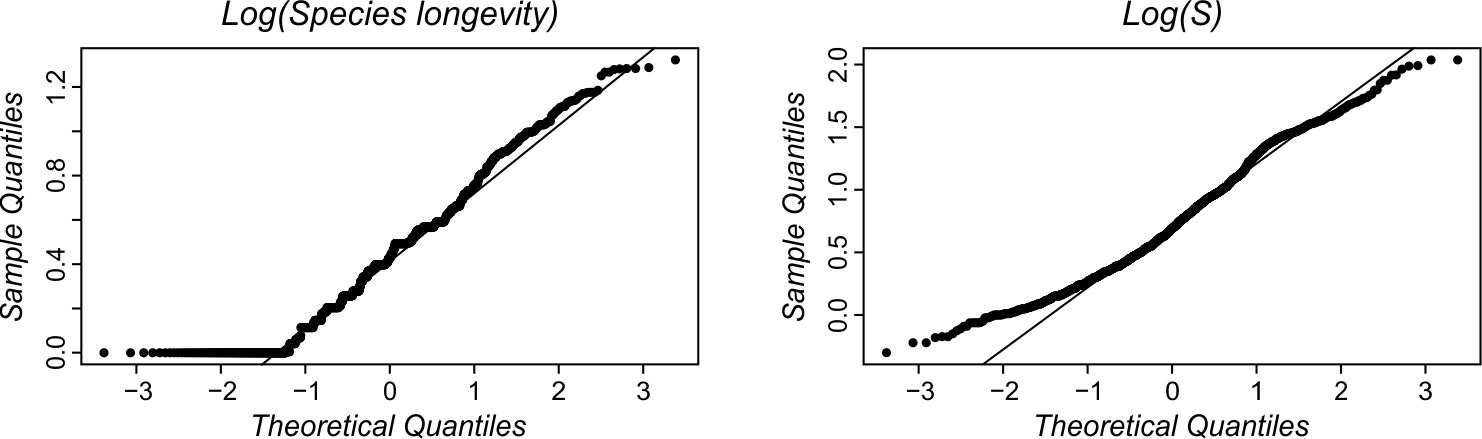


**Table S1.6** **Results of the linear regressions**

|  | **Slope b_1_** | **SE** | **Intercept b_0_** | **SE** | **P** | **R^2^** |
| --- | --- | --- | --- | --- | --- | --- |
| Size vs. longevity | 0.272 | 0.040 | 0.619 | 0.021 | 0.000 | 0.033 |

**Fig. S1.5** Plot of shell size versus species longevity


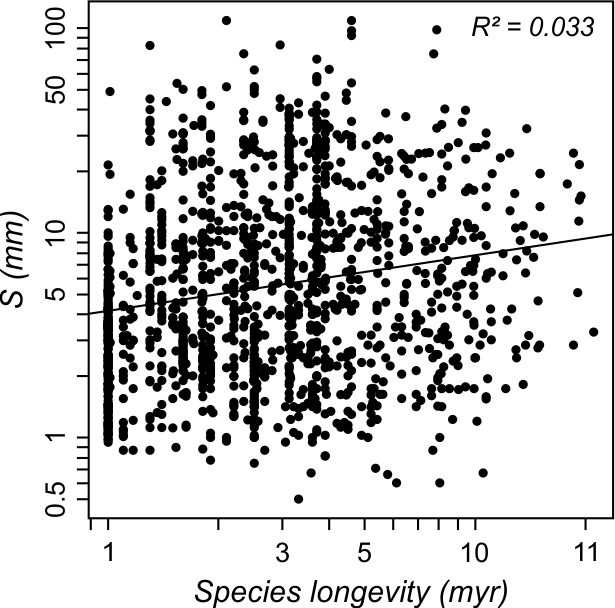


***4. Linear regressions of size measures with lake age***

**Table S1.7** Data for the linear regressions of size measures with lake age

| **Lake** | **Horizon [Ma]** | **Lake age [Ma]** | ***S_min_*** | ***S_max_*** | ***S_mean_*** | ***S_range_*** |
| --- | --- | --- | --- | --- | --- | --- |
| Caspia | 0 | 0.88 | -0.0089 | 0.9911 | 0.5466 | 0.9454 |
| Dacia 1 | 8 | 0.6 | 0.1712 | 1.4710 | 0.9646 | 1.4487 |
| Dacia 2 | 6 | 2.6 | 0.1761 | 1.9917 | 1.3139 | 1.9850 |
| Dacia 3 | 4 | 4.6 | -0.2218 | 1.6827 | 1.1580 | 1.6773 |
| Drniš | 15.5 | 0.2 | 0.0880 | 1.4606 | 0.8579 | 1.4418 |
| Metohia 1 | 5.5 | 0.54 | -0.0902 | 1.6127 | 1.2403 | 1.6040 |
| Metohia 2 | 3 | 3.04 | 0.1901 | 1.6087 | 1.1660 | 1.5919 |
| Nördlinger Ries | 14.4 | 0.6 | 0.4515 | 1.5085 | 1.0867 | 1.4687 |
| Ohrid | 0 | 1.5 | -0.0235 | 1.5880 | 0.7626 | 1.5773 |
| Pamvotis | 0 | 0.4 | 0.1461 | 1.6901 | 1.0608 | 1.6775 |
| Pannon 1 | 11 | 0.6 | -0.3010 | 2.0368 | 0.9975 | 2.0348 |
| Pannon 2 | 9 | 2.6 | -0.2218 | 2.0368 | 0.9908 | 2.0344 |
| Pannon 3 | 7 | 4.6 | -0.2218 | 1.8741 | 0.9889 | 1.8706 |
| Pannon 4 | 5 | 6.6 | -0.2218 | 1.8741 | 0.9768 | 1.8706 |
| Sinj | 15.5 | 2.5 | -0.0568 | 1.4606 | 0.7987 | 1.4472 |
| Steinheim | 13.8 | 1.2 | -0.0625 | 1.5085 | 0.7394 | 1.4967 |
| Trichonis | 0 | 2.6 | 0.0538 | 1.4163 | 0.8314 | 1.3970 |

**Note:** Lake age refers to the geological age of the lake at the respective stratigraphic horizon.

**Table S1.8** Shapiro-Wilk normality test

| **Parameter/size measure** | ***W*** | ***P*** |
| --- | --- | --- |
| Maximum size | 0.9267 | 0.1915 |
| Mean size | 0.9814 | 0.9688 |
| Minimum size | 0.944 | 0.3692 |
| Size range | 0.9248 | 0.1780 |
| log(Lake age) | 0.9488 | 0.4376 |

**Table S1.9** Results of the linear regressions

| **Size measure** | **Slope b_1_** | **SE** | **Intercept b_0_** | **SE** | **P** | **R^2^** |
| --- | --- | --- | --- | --- | --- | --- |
| Size range | 0.219 | 0.156 | 1.590 | 0.069 | 0.057 | 0.116 |
| Maximum size | 0.207 | 0.151 | 1.606 | 0.067 | 0.189 | 0.112 |
| Minimum size | -0.185 | 0.108 | 0.018 | 0.048 | 0.109 | 0.162 |
| Mean size | 0.057 | 0.117 | 0.961 | 0.052 | 0.632 | 0.016 |

***5. Linear regressions of size measures with species richness, using the subsampled dataset***

**Table S1.10** Subsampled dataset for the linear regressions of size measures with species richness

| **Lake** | **species richness** | ***S_min_*** | ***S_max_*** | ***S_mean_*** | ***S_range_*** |
| --- | --- | --- | --- | --- | --- |
| Bresse | 64 | 0.0857 | 1.5880 | 0.9198 | 1.5742 |
| Caspia | 92 | -0.0089 | 0.9911 | 0.5466 | 0.9454 |
| Dacia 1 | 27 | 0.1712 | 1.4710 | 0.9646 | 1.4487 |
| Dacia 2 | 57 | 0.1761 | 1.9917 | 1.3139 | 1.9850 |
| Dacia 3 | 122 | -0.2218 | 1.6827 | 1.1580 | 1.6773 |
| Drniš | 43 | 0.0880 | 1.4606 | 0.8579 | 1.4418 |
| Gacko | 12 | 0.3333 | 1.1456 | 0.7297 | 1.0729 |
| Granada | 21 | 0.0485 | 1.4163 | 0.9808 | 1.3972 |
| Groisenbach | 12 | 0.2559 | 1.0997 | 0.6721 | 1.0325 |
| Kosovo | 22 | -0.0902 | 1.5396 | 1.2648 | 1.5293 |
| Kupres | 23 | 0.0880 | 1.1456 | 0.7514 | 1.1058 |
| Le Locle | 18 | 0.2096 | 1.5085 | 1.0472 | 1.4861 |
| Metohia 1 | 36 | -0.0902 | 1.6127 | 1.2403 | 1.6040 |
| Metohia 2 | 22 | 0.1901 | 1.6087 | 1.1660 | 1.5919 |
| Nördlinger Ries | 6 | 0.4515 | 1.5085 | 1.0867 | 1.4687 |
| Ohrid | 68 | -0.0235 | 1.5880 | 0.7626 | 1.5773 |
| Pamvotis | 25 | 0.1461 | 1.6901 | 1.0608 | 1.6775 |
| Pannon 1 | 219 | -0.3010 | 2.0368 | 0.9975 | 2.0348 |
| Pannon 2 | 250 | -0.2218 | 2.0368 | 0.9908 | 2.0344 |
| Pannon 3 | 152 | -0.2218 | 1.8741 | 0.9889 | 1.8706 |
| Pannon 4 | 147 | -0.2218 | 1.8741 | 0.9768 | 1.8706 |
| Randeck Maar | 4 | 0.4515 | 1.5085 | 1.1261 | 1.4687 |
| Sinj | 58 | -0.0568 | 1.4606 | 0.7987 | 1.4472 |
| Slavonia | 165 | 0.1505 | 1.5731 | 1.0955 | 1.5563 |
| Sofia | 6 | 0.3495 | 1.5000 | 1.1673 | 1.4682 |
| Sostanj | 9 | 0.2593 | 1.0792 | 0.6803 | 1.0079 |
| Steinheim | 42 | -0.0625 | 1.5085 | 0.7394 | 1.4967 |
| Transylvania | 78 | 0.1901 | 1.4856 | 0.8639 | 1.4631 |
| Trichonis | 23 | 0.0538 | 1.4163 | 0.8314 | 1.3970 |

**Note:** Species richness values also include species where no size information is available.

**Table S1.11** Shapiro-Wilk normality test

| **Parameter/size measure** | ***W*** | ***P*** |
| --- | --- | --- |
| Maximum size | 0.9348 | 0.0733 |
| Mean size | 0.9788 | 0.8080 |
| Minimum size | 0.9691 | 0.5364 |
| Size range | 0.9308 | 0.0577 |
| log(Species richness) | 0.9741 | 0.6746 |

**Table S1.12** Results of the linear regressions

| **Size measure** | **Slope b_1_** | **SE** | **Intercept b_0_** | **SE** | **P** | **R^2^** |
| --- | --- | --- | --- | --- | --- | --- |
| Size range | 0.336 | 0.094 | 0.983 | 0.153 | 0.001 | **0.322** |
| Maximum size | 0.309 | 0.089 | 1.049 | 0.146 | 0.002 | **0.307** |
| Minimum size | -0.346 | 0.046 | 0.616 | 0.075 | 0.000 | **0.676** |
| Mean size | -0.010 | 0.078 | 0.974 | 0.127 | 0.899 | 0.001 |

***6. Results of resampling***

**Table S1.13** Estimates of the regression slopes of size measures on area and endemism. The statistical significance of the regression slopes was tested using the resampling technique as described in the main text.

| **Size measure** | **Variable** | **Observed Slope b_1_** | **Parametric P-value** | **P-value of**  **resampling test** |
| --- | --- | --- | --- | --- |
| Maximum size* | Area | 0.116 | <0.001 | 0.925 |
| Minimum size | Area | -0.071 | 0.007 | 0.248 |
| Size range* | Area | 0.124 | 0.001 | 0.970 |
| Minimum size | Endemism | -0.003 | 0.033 | 0.052 |

The null model here is that the observed correlations are independent of species richness. Given that all resampled *P* > 0.05, the null hypothesis cannot be rejected and the relationships of abovementioned size measures with area and degree of endemism are independent of species richness (compare Roy & Marien, 2001). Regressions marked by an asterisk are based on the dataset excluding the Caspian Sea (see main text for details).

**REFERENCES**

Roy, K. & Martien, K.K. (2001) Latitudinal distribution of body size in north-eastern Pacific marine bivalves. *Journal of Biogeography*, **28**, 485-493.
