# Supplementary material for: Predictors of shell size in long‐lived lake gastropods
Source: J Biogeogr. 2016 Jul 21;43(10):2062–74. doi: 10.1111/jbi.12777 (PMC5042061; doi:10.1111/jbi.12777)
Supplement: Supplementary file 2 — Appendix S2 Beta‐Jaccard dissimilarity measure for pairwise comparisons. [file JBI-43-2062-s002.docx]

*Journal of Biogeography*

**SUPPORTING INFORMATION**

**Predictors of shell size in long-lived lake gastropods**

Thomas A. Neubauer*, Elisavet Georgopoulou, Mathias Harzhauser, Oleg Mandic, Andreas Kroh

**Appendix S2**

**Table S2.14** Results of the Beta-Jaccard dissimilarity measure for pairwise comparisons. In addition, mean dissimilarity value per lake is given.

|  | Groisenbach | Gacko | Kupres | Sofia | Le Locle | Nördlinger Ries | Randeck Maar | Steinheim | Granada | Bresse | Pamvotis | Trichonis | Drniš | Sinj | Slavonia | Pannon | Kosovo | Metohia | Ohrid | Dacia | Transylvania | Caspia | **mean** |
| --- | --- | --- | --- | --- | --- | --- | --- | --- | --- | --- | --- | --- | --- | --- | --- | --- | --- | --- | --- | --- | --- | --- | --- |
| Groisenbach |  |  |  |  |  |  |  |  |  |  |  |  |  |  |  |  |  |  |  |  |  |  | 0.540 |
| Gacko | 0.500 |  |  |  |  |  |  |  |  |  |  |  |  |  |  |  |  |  |  |  |  |  | 0.528 |
| Kupres | 0.333 | 0.500 |  |  |  |  |  |  |  |  |  |  |  |  |  |  |  |  |  |  |  |  | 0.551 |
| Sofia | 0.714 | 0.625 | 0.875 |  |  |  |  |  |  |  |  |  |  |  |  |  |  |  |  |  |  |  | 0.638 |
| Le Locle | 0.667 | 0.444 | 0.667 | 0.625 |  |  |  |  |  |  |  |  |  |  |  |  |  |  |  |  |  |  | 0.502 |
| Nördlinger Ries | 0.333 | 0.500 | 0.333 | 0.714 | 0.500 |  |  |  |  |  |  |  |  |  |  |  |  |  |  |  |  |  | 0.500 |
| Randeck Maar | 0.600 | 0.714 | 0.833 | 0.500 | 0.714 | 0.600 |  |  |  |  |  |  |  |  |  |  |  |  |  |  |  |  | 0.728 |
| Steinheim | 0.625 | 0.556 | 0.778 | 0.571 | 0.556 | 0.625 | 0.667 |  |  |  |  |  |  |  |  |  |  |  |  |  |  |  | 0.584 |
| Granada | 0.429 | 0.375 | 0.429 | 0.571 | 0.375 | 0.167 | 0.667 | 0.500 |  |  |  |  |  |  |  |  |  |  |  |  |  |  | 0.445 |
| Bresse | 0.500 | 0.455 | 0.500 | 0.600 | 0.455 | 0.500 | 0.800 | 0.545 | 0.400 |  |  |  |  |  |  |  |  |  |  |  |  |  | 0.413 |
| Pamvotis | 0.700 | 0.636 | 0.818 | 0.500 | 0.500 | 0.700 | 0.750 | 0.444 | 0.600 | 0.500 |  |  |  |  |  |  |  |  |  |  |  |  | 0.565 |
| Trichonis | 0.600 | 0.667 | 0.727 | 0.556 | 0.545 | 0.600 | 0.778 | 0.333 | 0.500 | 0.273 | 0.300 |  |  |  |  |  |  |  |  |  |  |  | 0.490 |
| Drniš | 0.375 | 0.333 | 0.375 | 0.667 | 0.333 | 0.375 | 0.750 | 0.600 | 0.250 | 0.200 | 0.545 | 0.455 |  |  |  |  |  |  |  |  |  |  | 0.409 |
| Sinj | 0.500 | 0.455 | 0.500 | 0.727 | 0.455 | 0.500 | 0.800 | 0.667 | 0.400 | 0.333 | 0.615 | 0.538 | 0.200 |  |  |  |  |  |  |  |  |  | 0.481 |
| Slavonia | 0.545 | 0.500 | 0.545 | 0.636 | 0.500 | 0.545 | 0.818 | 0.583 | 0.455 | 0.091 | 0.538 | 0.333 | 0.273 | 0.385 |  |  |  |  |  |  |  |  | 0.435 |
| Pannon | 0.750 | 0.650 | 0.750 | 0.800 | 0.650 | 0.750 | 0.900 | 0.700 | 0.700 | 0.500 | 0.600 | 0.550 | 0.600 | 0.500 | 0.450 |  |  |  |  |  |  |  | 0.607 |
| Kosovo | 0.500 | 0.600 | 0.286 | 0.778 | 0.600 | 0.500 | 0.875 | 0.818 | 0.556 | 0.455 | 0.750 | 0.667 | 0.500 | 0.583 | 0.500 | 0.650 |  |  |  |  |  |  | 0.556 |
| Metohia | 0.636 | 0.692 | 0.500 | 0.833 | 0.583 | 0.636 | 0.909 | 0.769 | 0.667 | 0.333 | 0.714 | 0.538 | 0.500 | 0.571 | 0.250 | 0.500 | 0.300 |  |  |  |  |  | 0.545 |
| Ohrid | 0.600 | 0.667 | 0.600 | 0.700 | 0.545 | 0.444 | 0.778 | 0.500 | 0.500 | 0.273 | 0.455 | 0.200 | 0.455 | 0.538 | 0.333 | 0.550 | 0.545 | 0.417 |  |  |  |  | 0.493 |
| Dacia | 0.615 | 0.571 | 0.615 | 0.692 | 0.462 | 0.615 | 0.846 | 0.733 | 0.538 | 0.357 | 0.600 | 0.533 | 0.385 | 0.357 | 0.286 | 0.350 | 0.462 | 0.357 | 0.533 |  |  |  | 0.503 |
| Transylvania | 0.636 | 0.583 | 0.636 | 0.600 | 0.455 | 0.500 | 0.800 | 0.667 | 0.400 | 0.333 | 0.500 | 0.417 | 0.364 | 0.333 | 0.250 | 0.500 | 0.583 | 0.462 | 0.417 | 0.357 |  |  | 0.474 |
| Caspia | 0.500 | 0.625 | 0.500 | 0.667 | 0.625 | 0.500 | 0.800 | 0.571 | 0.333 | 0.600 | 0.667 | 0.556 | 0.500 | 0.600 | 0.636 | 0.800 | 0.625 | 0.727 | 0.700 | 0.692 | 0.600 |  | 0.579 |
| Šoštanj | 0.750 | 0.500 | 0.571 | 0.714 | 0.286 | 0.571 | 0.833 | 0.625 | 0.429 | 0.500 | 0.556 | 0.600 | 0.375 | 0.500 | 0.545 | 0.750 | 0.667 | 0.636 | 0.600 | 0.615 | 0.500 | 0.500 | 0.549 |
