# Supplementary material for: Predictors of shell size in long‐lived lake gastropods
Source: J Biogeogr. 2016 Jul 21;43(10):2062–74. doi: 10.1111/jbi.12777 (PMC5042061; doi:10.1111/jbi.12777)
Supplement: Supplementary file 3 — Appendix S3 Minimum size versus endemism for gastropod families. [file JBI-43-2062-s003.docx]

*Journal of Biogeography*

**SUPPORTING INFORMATION**

**Predictors of shell size in long-lived lake gastropods**

Thomas A. Neubauer*, Elisavet Georgopoulou, Mathias Harzhauser, Oleg Mandic, Andreas Kroh

**Appendix S3**

**Fig. S3.6** Minimum size versus degree of endemism for the ten most diverse families.


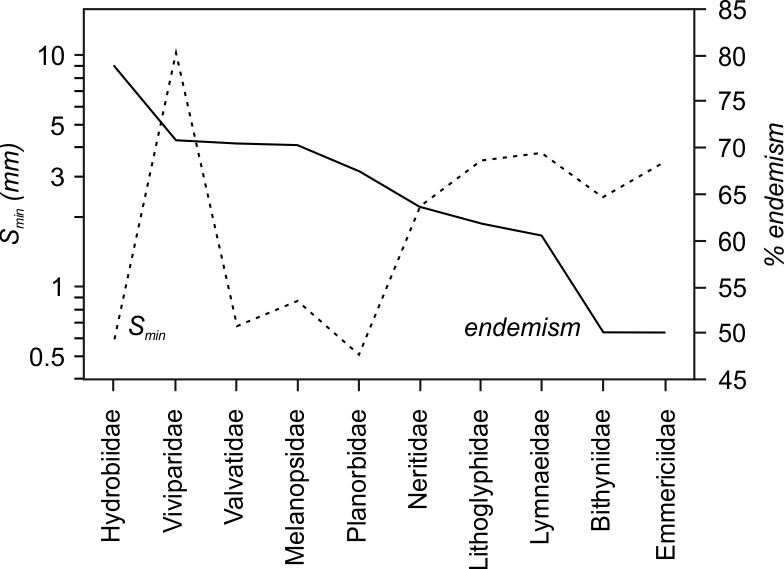


Note that no significant relation between degree of endemism per gastropod family and its minimum shell size is detected.
